# Interventional Dilemma in a Young Woman With Spontaneous Coronary Artery Dissection Who Presented With High-Risk Non-ST-Elevation Myocardial Infarction (NSTEMI) Progressing to ST-Elevation Myocardial Infarction (STEMI)

**DOI:** 10.7759/cureus.23983

**Published:** 2022-04-09

**Authors:** Maleeha Saleem, Shazia M Shah, Justin Fox

**Affiliations:** 1 Internal Medicine, Saint Francis Medical Center, Trenton, USA; 2 Cardiology, Saint Francis Medical Center, Trenton, USA

**Keywords:** non-st-elevation mi, intramural hematoma, optical coherence tomography, intravascular ultrasound, st-elevation mi, spontaneous coronary artery dissection

## Abstract

Spontaneous coronary artery dissection (SCAD) is an infrequent presentation of acute myocardial infarction in young women and denotes the non-atherosclerotic separation of the coronary artery wall. Precipitating causes include fibromuscular dysplasia, postpartum hormonal changes, multiparity, connective tissue diseases like Marfan syndrome, autoimmune conditions, and hormonal therapy. It is often underdiagnosed due to a low index of suspicion based on age and gender bias as well as knowledge about different angiographic variants in SCAD. Intracoronary imaging with optical coherence tomography (OCT) or intravascular ultrasound (IVUS) is used for patients where coronary angiography fails to secure a diagnosis to increase the diagnostic yield. The mainstay of stable SCAD is conservative management. However, there are no definitive guidelines due to limited clinical experience. Treatment involving percutaneous coronary intervention (PCI), coronary artery bypass grafting (CABG), fibrinolytic therapy, and mechanical hemodynamic support should be individualized depending upon clinical presentation, type, and extent of dissection, hemodynamic instability, critical anatomy involvement, and the extent of ischemic myocardium. We are presenting a case of a young female who presented with non-ST-elevation myocardial infarction (NSTEMI) that progressed to ST-elevation myocardial infarction (STEMI). A coronary angiogram showed a tortuous left anterior descending artery (LAD) with a distal 100% occlusion due to SCAD. PCI was attempted but the guidewire could not be navigated intraluminally past the occlusion. CABG was not pursued due to the distal location of the occlusion and lack of visualization of the distal vessel. Our case provides a useful learning opportunity for physicians who may come across similar clinical presentations. In patients with high-risk features of SCAD who are deemed inoperable, timely and appropriate medical management may be a useful alternative for PCI/CABG and the recurrence rates of SCAD are very low.

## Introduction

Acute coronary syndrome (ACS) is most often caused by atherosclerotic plaque rupture with subsequent thrombus formation in patients with risk factors of coronary artery disease (CAD). However, other causes of acute myocardial infarction (AMI), such as coronary vasospasm and aortic dissection, should be considered [1]. In the differential diagnosis of ACS, the phenomenon of spontaneous coronary artery dissection (SCAD) is important to consider, particularly in young females with no known CAD risk factors [2]. It is imperative to establish the diagnosis of SCAD as the risk of complications including sudden cardiac death and recurrent dissection remains very high in missed or untreated cases. In the absence of hemodynamic instability, ongoing ischemic chest pain, and/or left main stem involvement requiring percutaneous coronary intervention (PCI), most cases are not intervened upon and are treated with pharmacotherapy only [3]. PCI may be considered with an increased risk as compared to other ACS subtypes due to the risk of entering the false lumen and either propagating the dissection or worsening an intramural hematoma [2]. Our case describes a 35-year-old female who presented with non-ST-elevation myocardial infarction (NSTEMI) and high-risk features including ongoing ischemia that progressed to ST-elevation myocardial infarction (STEMI) after an unsuccessful attempt at PCI. This case is an important learning tool for physicians that experience similar management challenges and how appropriate medical therapy can serve as a safe alternative to PCI/coronary artery bypass grafting (CABG) in high-risk patients.

## Case presentation

**Figure 1:**
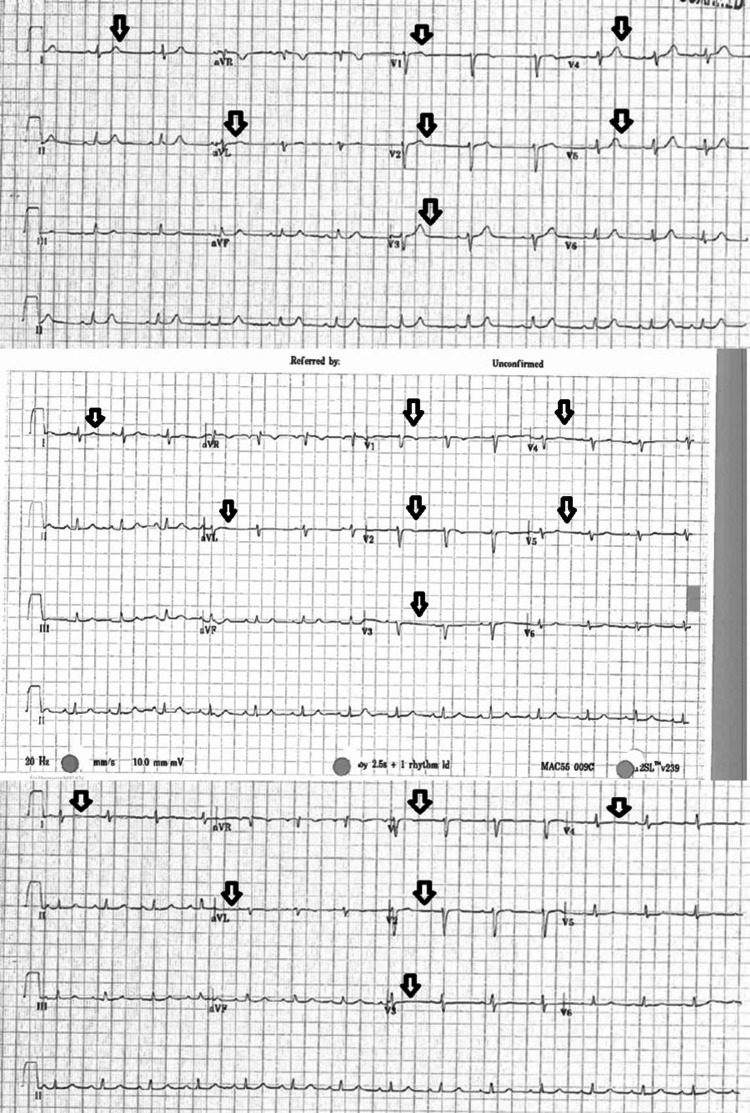
Serial EKGs showing T wave flattening and then hyperacute T waves concerning for anterolateral ischemia.

A 35-year-old Caucasian woman without a prior history of chest pain or ischemic heart disease presented to the emergency department with substernal chest pain for one day. The chest pain was pressure-like in nature, intermittent with subsequent progression to being constant, and 10 out of 10 in intensity with radiation to both shoulders and left jaw. There were no reported aggravating or relieving factors. There was associated diaphoresis and nausea but no shortness of breath, palpitations, dizziness, or vomiting. Past medical history was significant for obesity, fibromyalgia, and anxiety. Surgical history included a recent cesarean section three months prior, tubal ligation, and surgical resection of ovarian teratoma. Family history was significant for premature coronary artery disease in grandmother. Initial vitals showed a blood pressure of 115/79 mmHg with a heart rate of 89 beats per minute. On physical examination, the carotids were +2 bilaterally and there was no jugular venous distension. On auscultation of the heart, there was a regular rate and rhythm, normal S1 and S2, and no S3 or S4. The point of maximal impulse (PMI) was not displaced and there were no murmurs, rubs, or gallops. She received a full dose of aspirin and nitroglycerin, which helped in reducing the intensity of chest pain. Her initial electrocardiogram (EKG) was normal, but repetitive EKGs showed evolving changes of T wave flattening and subsequently hyperacute T waves in the anterior and lateral leads concerning for ischemia, as shown in Figure 1. Initial transthoracic echocardiogram (TTE) showed a left ventricular ejection fraction (LVEF) of about 40-45% with akinetic apical septal, apical anterior, apical lateral, and apical inferior wall segments.

**Figure 2:**
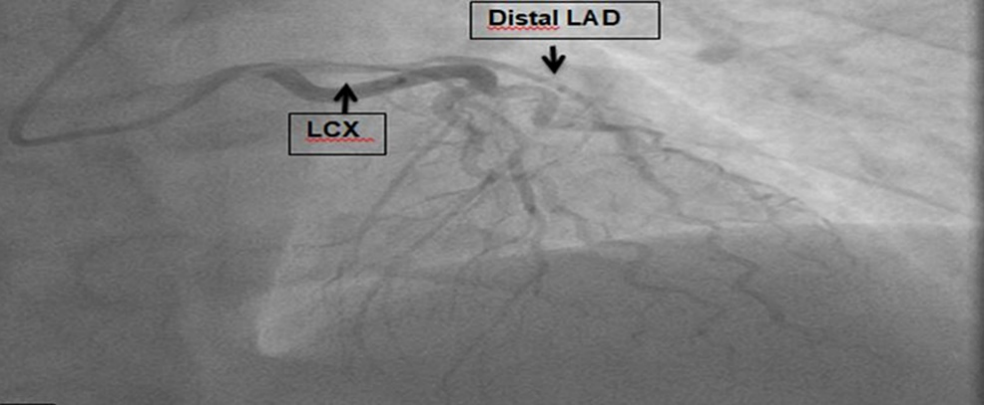
Coronary angiogram showing a severe narrowing in distal LAD with vasospasm due to coronary dissection. LAD: left anterior descending artery; LCX: left circumflex.

**Figure 3:**
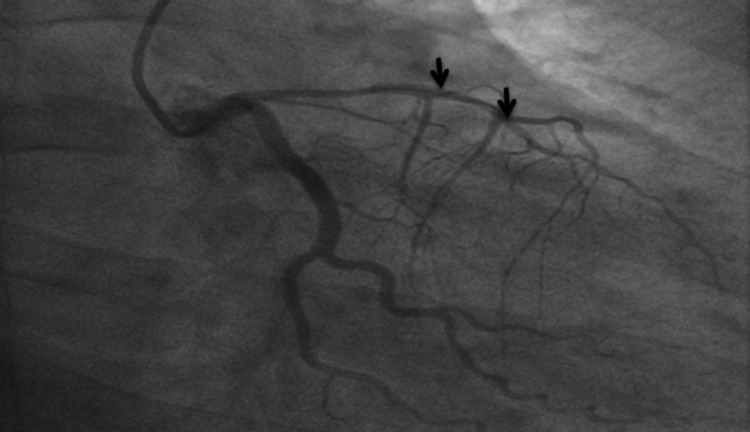
Coronary angiogram showing failed attempts to wire the occluded distal left anterior descending artery and first diagonal due to false lumen as indicated by arrows.

Initial troponin was 0.43, which peaked at 16. Under the impression that the patient is having acute NSTEMI, she was taken for emergent cardiac catheterization with possible PCI. The following angiographic findings were noted: (i) the left main was angiographically normal and bifurcated into the LAD and left circumflex (LCX). (ii) The LAD is a large vessel that is wrapped around the apex of the heart. It gave several septal and diagonal branches. The LAD was diffusely narrowed at approximately 70% proximal to the mid-vessel and the diagnosis of SCAD was made based on angiographic appearance. (iii) The diagonal branch was totally occluded, as was the distal LAD. (iv) LCX is a large vessel. LCX and its branches were angiographically normal. (v) The right coronary artery (RCA) is a medium-caliber vessel, and it was angiographically normal. Intervention to the LAD was undertaken. The decision was made to attempt to wire the distal LAD to attempt to reperfuse the apex if possible. Bivalirudin bolus and drip were administered. The left main was approached with an extra backup (EBU) 3.5 guide catheter and a Terumo Runthrough wire (Terumo Corporation, Shibuya City, Tokyo) was advanced into the vessel. The tip of the wire was unable to navigate the occlusion of the distal LAD easily. When the wire tip eventually passed, it appeared to be taking a subintimal course and there was a concern that the wire tip was going in a false lumen. This also occurred when a second attempt to wire the totally occluded diagonal branch was made. Further attempts were not pursued because of the possible risk of harm as the wire did not appear to be coursing intraluminally to the distal vessels and the vessel was left unchanged from the diagnostic angiogram appearance, as shown in Figures 2, 3.

**Figure 4:**
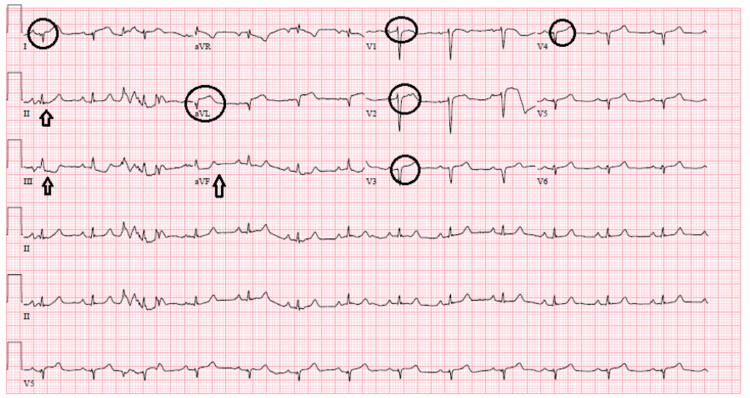
ST-segment elevations in 1, avL, v1, v2, v3, and v4, as shown by circles, and reciprocal ST-segment depressions in II, III, and avF, as shown by arrows. avL: augmented voltage left arm; avF: augmented voltage foot.

After discussion with cardiothoracic surgery, CABG was not considered an appropriate option as the distal vessel was occluded and not visualized. The decision was made to keep the patient in the ICU for conservative management. About four hours post-catheterization, a repeat EKG due to a sudden worsening of ongoing chest pain demonstrated ST elevations in anterior and lateral chest leads and reciprocal ST depressions in inferior leads concerning for anterolateral STEMI, as shown in Figure 4.

However, given the prior unsuccessful attempts at intervention and ongoing symptoms, along with the distal vessel occlusion, the patient was started on an unfractionated heparin drip along with aspirin, clopidogrel, carvedilol, and lisinopril. A multi-disciplinary team involving cardiologists and CT surgery decided that the benefits of therapeutic heparinization to minimize intraluminal thrombosis outweighed the risks of worsening intraluminal hematoma. Her chest pain significantly improved over the next few days after the start of therapeutic heparinization. Repeat echocardiogram and diagnostic workup of fibromuscular dysplasia were recommended as an outpatient but the patient was lost to follow up.

## Discussion

SCAD is a non-traumatic and non-atherosclerotic cause of ACS in young females [3]. It is a rare but notable etiology of ACS in patients with no known risk factors of CAD [4]. The reported prevalence is approximately 0.07-1% but the true prevalence is believed to be higher due to underdiagnosed cases [5]. The majority of diagnosed cases are in women and the reported mean age is less than 50 years [6]. Symptoms range from chest pain, dyspnea, palpitations, sudden cardiac arrest, or hemodynamic instability leading to cardiogenic shock [2]. Presentations may vary from unstable angina to NSTEMI to STEMI [5]. The etiology varies from idiopathic to precipitated causes including coronary vessel spasms, fibromuscular dysplasia, autoimmune diseases, connective tissue disorders, hormonal therapy, and those associated with potential stressors such as postpartum states, intense exercise or emotional stress, labor, and delivery [5,6].

Coronary angiography remains the mainstay of diagnosis. The Yip-Saw classification is adapted to classify SCAD into three types based on angiographic features [7]. Common findings include intimal flap, two separate communicating lumens, multiple dissecting lines, and coronary aneurysm communicating with the lumen [8]. LAD involvement is more common in females as opposed to RCA involvement [9]. Diagnostic accuracy can be increased with the use of both intravascular ultrasound (IVUS) and optical coherence tomography (OCT) [10].

To date, there are no established guidelines for the management of SCAD in the literature [1]. Management should be individualized depending upon clinical presentation and the concomitant angiographic findings and range from conservative medical management to emergent revascularization with PCI or CABG [11]. Revascularization is a feasible option in patients with unstable hemodynamics, arrhythmias such as ventricular tachycardia (VT) and/or fibrillation, recurrent or persistent chest pain indicative of ongoing ischemia, and left main CAD [11]. Recent observational studies suggested that medical management is recommended in stable SCAD patients as there have been reported high rates of angiographic healing of coronary dissection and favorable short-term outcomes [12].

In our case, we decided to perform emergent PCI as the patient was having persistent chest pain and significant EKG changes, elevated troponins, and acute wall motion abnormalities on TTE. Coronary revascularization is extremely challenging due to technical difficulties in passing guidewire into the true lumen and the risk of extension of dissection by the catheter-based procedure [3]. Stenting is associated with numerous complications including stent thrombosis, in-stent restenosis, and displacement of intramural hematoma potentially compromising a large area of the myocardium [5]. Strategies to overcome this involve either the use of long or multiple stents covering the whole dissected portion of the artery or stenting the entry point of dissection. This sometimes requires precise localization by OCT [13]. We were able to diagnose SCAD based on angiographic appearance but multiple attempts at stenting failed and the use of OCT or IVUS was not indicated as narrowing was diffuse. In patients with long and diffuse dissection, cutting balloon angioplasty to fenestrate intima is a useful alternative; however, it is associated with a risk of coronary perforation [14].

There was a technical difficulty in advancing the guidewire into the true lumen after multiple attempts and further attempts were not made due to the risk of accessing the false lumen. A multi-disciplinary team involving cardiothoracic surgeons and cardiologists also concluded that CABG was not a feasible option as distal vasculature was not visible. After unsuccessful intervention to reopen the distal LAD and occluded diagonal and subsequent progression to STEMI, our team decided that anticoagulation with heparin drip can be a safe option in SCAD by carefully weighing the risk of intramural hematoma versus the benefit of reducing the thrombus burden. So, our patient with high-risk SCAD was medically managed. Therapeutic anticoagulation was the cornerstone of management in our case. Its use is best reserved on a case-by-case basis until further research is done. Though our patient did not receive any stenting or angioplasty, due to her high-risk clinical presentation and progressive EKG changes, dual antiplatelet therapy was started and recommended to be continued for at least one year. She was also started on a beta-blocker that has been shown to prevent recurrent SCAD [2]. Statins are usually not the first-line treatment of SCAD as the lesions by definition are non-atherosclerotic and statins are associated with a higher rate of SCAD recurrence [15]. She also received lisinopril as a part of conservative management of SCAD due to ischemic cardiomyopathy [6]. Our patient significantly improved with anticoagulation along with close hemodynamic monitoring, thus suggesting anticoagulation is an important part of conservative management. Is it a safer alternative to stenting in SCAD? Further investigations and ultimately a clinical trial would be needed to answer this question.

## Conclusions

SCAD presenting as high-risk NSTEMI should be emergently managed with coronary revascularization as the risk of sudden cardiac death and progression of dissection and fatal ventricular arrhythmias is very high. In patients who have technical difficulty with stenting or angioplasty, successful revascularization rates can be increased with the use of IVUS and OCT. However, in our patient, with failed attempts at PCI and repeat EKG showing ST elevations, therapeutic heparinization was pursued by the multidisciplinary team after carefully ruling out risks. This case highlights anticoagulation as an important aspect of conservative management in high-risk ACS patients on a case-by-case basis where multiple attempts at PCI have been unsuccessful and CABG is not feasible.
